# Prevalence and characterization of Panton-Valentine leukocidin-positive *Staphylococcus aureus* in bovine milk in Jabalpur district of Madhya Pradesh, India

**DOI:** 10.14202/vetworld.2018.316-320

**Published:** 2018-03-16

**Authors:** Neeraj Shrivastava, Varsha Sharma, Arpita Shrivastav, Anju Nayak, Ajay Kumar Rai

**Affiliations:** 1 Department of Veterinary Microbiology, College of Veterinary Science and Animal Husbandry, Jabalpur, Madhya Pradesh, India; 2 Department of Veterinary Pharmacology and Toxicology, College of Veterinary Science and Animal Husbandry, Jabalpur, Madhya Pradesh, India

**Keywords:** bovine milk, methicillin-resistant *Staphylococcus aureus*, Panton-Valentine leukocidin

## Abstract

**Aim:**

The study aimed to investigate the Panton-Valentine leukocidin (PVL)-positive *Staphylococcus aureus* in bovine milk due to its public health significance.

**Materials and Methods:**

A total of 400 milk samples of bovines taken from different dairy farms and outlets of Jabalpur were screened for the *S. aureus* and methicillin-resistant *S. aureus* (MRSA). The strains were tested for the PVL gene and antimicrobial sensitivity toward 10 different classes of antimicrobial agents. The PVL-positive *S. aureus* strains were further characterized by staphylococcal protein A or *spa* typing.

**Result:**

The prevalence of PVL-positive *S. aureus* was 10.53%. All the isolates positive for the PVL were resistant to methicillin, while the methicillin-sensitive *S. aureus* isolates were negative for the PVL. Five different *spa* types were found.

**Conclusion:**

The presence of PVL-positive MRSA in bovine milk close to consumer poses a potential public health risk to the community.

## Introduction

*Staphylococcus aureus* causes mild skin and soft tissue infections in humans and domestic animals to serious diseases like necrotizing pneumonia in humans and economically important mastitis in dairy ruminants. *S. aureus* is considered as the most ubiquitous and dangerous human and veterinary pathogens, for both its virulence and its ability to develop antibiotic resistance [1-3].

Among the large array of virulence factors, leukotoxins constitute a family of pore-forming toxins that, by targeting phagocytic cells, are likely to interfere with immune defenses and contribute to the severity of staphylococcal infections. Members of this toxin family present clinical association and epidemiological distribution with particular human diseases or with mastitis of dairy ruminants. In humans, the presence of the Panton-Valentine leukocidin (PVL) gene is associated with increased disease severity in *S. aureus* infections ranging from cutaneous infections to chronic osteomyelitis and severe necrotizing pneumonia with a high mortality rate [2]. The PVL toxin is commonly associated with community-acquired methicillin-resistant *S. aureus* (MRSA).

Mastitis-causing *S. aureus* strains in dairy animals are equipped with several leukotoxins: All strains possess the γ-hemolysin genes (*hl*g), most of them possess the genes for *LukE/*D, and 10-50% of isolates from bovine mastitis possess the genes for *LukM/F’-PV*. *LukM/F’-PV* is highly active on ruminant neutrophils and is the most cytotoxic leukotoxin on bovine neutrophils [3] PVL is weakly active on bovine neutrophils but strongly active on human polymorphonuclear cells. The PVL-positive *S. aureus* strains have been reported as a cause of mastitis from several countries [3,4].

The PVL is a well-recognized toxin produced by *S. aureus* strains and is a recognized marker of virulent *S. aureus* strains. The diffusion of PVL genes to different MRSA lineages is mediated by PVL bacteriophages. PVL-producing *S. aureus* strains have a notable geographic variation in prevalence. This study was aimed to study the prevalence of PVL-positive strains in Jabalpur area. The use of raw milk in the study provides an opportunity to study the prevalence at human-bovine interface.

## Materials and Methods

### Ethical approval

The authors declare that the research was conducted with prior approval from the Institutional Animal Ethics Committee, College of Veterinary Science and A.H., Jabalpur, Madhya Pradesh.

### Sample size calculation and collection of milk sample

As no prior information was available on the prevalence, the sample size was calculated based on a prevalence of 50% with an accuracy of ±5% at a 95% confidence level. Thus, a sample size of 384~400 samples was targeted [5].

A total of 400 milk samples were collected from the dairy farms and dairy outlets located in and around Jabalpur city (Table-1). The milking animals in the dairy farms were screened for mastitis using California Mastitis Test (CMT). The quarter or composite milk from dairy cattle and buffaloes with CMT score ≥1 or more [6] or the pooled, raw milk samples (10 ml) collected from the dairy outlets were collected in sterile tubes transported in ice and stored at −20°C till further use.

**Table-1:** Details of the milk samples collected for the study.

Species	Milk		Total

	Quarter/composite	Pooled	
Cow	85	45	130
Buffalo	103	167	270
Total	188	212	400

### Identification of PVL-positive *S. aureus* in bovine milk

As *S. aureus* shows heteroresistance mechanism in case of methicillin resistance, the 400 milk samples were screened in parallel for the *S. aureus* and MRSA [7].

The milk samples were subjected to one freeze-thaw cycle and inoculated into Mueller-Hinton broth (HiMedia) with 6.5% sodium chloride (NaCl) and incubated at 35 °C for 16-20 h.

### Isolation of *S. aureus* from milk

One loopful of this first pre-enrichment culture was then inoculated into Mannitol salt agar and incubated at 35°C for 16-20 h. Cells expressing heteroresistance grow more slowly than the methicillin-susceptible population and may be missed at temperatures above 35°C [8]. The single colony with typical morphology on the Mannitol salt agar plate was then streaked onto a Baird–Parker agar plate with egg yolk tellurite supplement (HiMedia) and incubated at 35°C for 18-24 h. The colony morphology (size, coloration, and lecithinase reaction) of the colonies obtained on the Baird–Parker agar plate was observed. The colonies were checked for purity by Gram’s staining for Gram’s reaction, cellular morphology, and arrangement in the Tryptone Soy Agar [1].

### Isolation of MRSA from milk

The 400 milk samples from bovines were screened for the MRSA using an additional selective enrichment step in Tryptone Soya Broth (HiMedia) with 4 mg/L cefoxitin (Sigma) and 75 mg/L aztreonam (Sigma) at 35°C for 16-20 h after the salt pre-enrichment [7]. A loopful of the inoculum from the previous step was streaked in HiChrome MeReSa agar plates with cefoxitin and methicillin supplement (HiMedia) and was incubated at 35°C for 16-20 h. Up to five blue-green colonies indicative of being MRSA were chosen and were streaked in Tryptone Soy Agar and incubated at 35°C for 16-20 h.

The presumptive *S. aureus*/MRSA isolates were phenotypically characterized using catalase test, Gram’s reaction, Voges–Proskauer test, clumping factor test, tube coagulase test, hemolysis in sheep blood agar, novobiocin and polymyxin B resistance, mannitol fermentation in Mannitol salt agar, and maltose fermentation in Purple agar [2].

### Isolation of genomic DNA from the *S. aureus*

The genomic DNA from the presumptive *S. aureus*/MRSA isolates was extracted using Instagene matrix (Biorad) as per manufacturer’s instruction.

### Multiplex polymerase chain reaction (PCR) for the detection of *spa, mecA*, and *lukF-PV genes*

The multiplex PCR protocol of EU Reference Laboratory–Antimicrobial Resistance (https://www.eurl-ar.eu/CustomerData/Files/Folders/21-protocols/279_pcr-spa-pvl-meca-mecc-sept12.pdf) for the detection of *spa, mecA*, and *lukF-PV* genes was followed. The primers for the *mecC* gene were not included in the protocol. The primers used in the study were purchased from Integrated DNA Technologies, USA (Table-2). The primer mix 1 and 2 for forward and reverse primers, respectively, were prepared by adding 25 μl of 100 μM of each primer in 900 μl of nuclease-free water (Thermo scientific). A 25 μl reaction was set up by adding 2 μl each of DNA template and primers mix 1 and 2 to the 12.5 μl of 2X DreamTaq™ Green PCR Master Mix (Thermo scientific). The cycling conditions (Veriti, ABI) were as follows: After an initial denaturation step at 94°C for 5 min, 30 cycles were performed, each consisting of 94°C for 30 sec, 59°C for 1 min, and 72°C for 1 min and followed by a final extension step at 72°C for 10 min.

**Table-2:** Details of the primers used in multiplex PCR (EURL-AR).

Gene	Primer sequence (5’-3’)	Size (bp)
*spa*	TAAAGACGATCCTTCGGTGAGC	180-600
	CAGCAGTAGTGCCGTTTGCTT	
*mecA*	TCCAGATTACAACTTCACCAGG	162
	CCACTTCATATCTTGTAACG	
*lukF-PV*	GCTGGACAAAACTTCTTGGAATAT	85
	GATAGGACACCAATAAATTCTGGATTG	

bp=Base pair, EURL-AR: EU reference laboratory–antimicrobial resistance, PCR=Polymerase chain reaction

**Figure-1:**
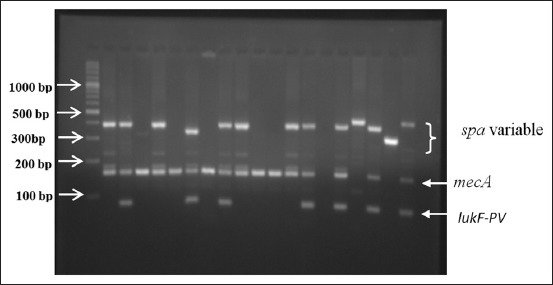
Agarose gel electrophoresis analysis showing multiplex polymerase chain reaction amplification products for the detection of the lukF-PV gene (85 bp). The Lanes 2, 6, 8, 13, 14, and 16 show the lukF-PV gene (85 bp) in the methicillin-resistant *Staphylococcus aureus* isolates. P = Positive control lukF-PV-positive *S. aureus*, N = Negative control.

The amplicons were separated on 1.5% agarose gel in 1X Tris-Borate ethylenediaminetetraacetic (TBE) buffer at 80 V for 90 min (Figure-1). The bands were documented using Gel documentation unit (Alpha Innotech). GeneRuler™ 100 base pair Plus DNA ladder, ready-to-use (Thermo Scientific) was used as a marker.

### *spa* typing of *lukF-PV*-positive *S. aureus* strains

The PCR products were sequenced by Sanger sequencing method (Genbank Accession no. MG821314-821319) at the SciGenom Labs, Cochin, India. The *spa* typing of the *S. aureus* was done as per user manual provided with the Ridom StaphType software version 2.2.1.

### Multidrug resistance profile of *lukF-PV*-positive *S. aureus*

The multidrug resistance profile of the *S. aureus* isolates was studied for 10 different class of antimicrobial agents by Kirby–Bauer disk diffusion assay according to the guidelines from the Clinical and Laboratory Standards Institute, 2012 (Table-3). *S. aureus* ATCC 25923 was used for quality control [8].

**Table-3:** Multidrug resistance profile of PVL-positive isolates.

Antimicrobial agent	Class	%S	%I	%R
Cefoxitin (30 µg)	β-lactams	0.0	0.0	100.0
Chloramphenicol (30 µg)	Phenicols	100.0	0.0	0.0
Ciprofloxacin (30 µg)	Fluoroquinolones	16.7	0.0	83.3
Clindamycin (2 µg)	Lincosamide	83.3	16.7	0.0
Co-trimoxazole (25 µg)	Folate inhibitors	16.7	0.0	83.3
Erythromycin (15 µg)	Macrolides	50.0	50.0	0.0
Gentamicin (10 µg)	Aminoglycosides	16.7	0.0	83.3
Linezolid (30 µg)	Oxazolidinones	100.0	0.0	0.0
Tetracycline (30 µg)	Tetracyclines	16.7	0.0	83.3
Pristinamycin (15 µg)	Streptogramin B	100.0	0.0	0.0

PVL=Panton-Valentine leukocidin

## Results and Discussion

*S. aureus* is a commensal present in the skin and nares of humans and bovines. It is also an important opportunistic pathogen with well-adapted host-specific lineages responsible for the disease in the respective host species [3]. In the recent and distant past, *S. aureus* has been shown to jump between human and bovine host in either direction [9,10]. The relationship between the virulence determinants and the clinical manifestation is complex. The pathogenicity of *S. aureus* infection is attributed to a wide array of virulence determinants rather than to any single one [11]. Nevertheless, PVL is strongly associated with life-threatening infections in humans.

In this study, the presence of PVL was detected in 10.53% of MRSA isolates, but none of the methicillin-sensitive *S. aureus* (MSSA) isolates carried this toxin (Table-4). The PVL gene was identified in more than 50% of isolates from bovine mastitis in Italy [12]. In China, the prevalence was reported to be 41.5% in the bovine isolates [13]. In India, the PVL gene was detected in 41.6 % of the *S. aureus* isolates from bovine milk; however, another study could not detect PVL gene in *S. aureus* isolates from bovine milk [14,15]. Few studies indicate that PVL in bovine isolates is a rare finding [11].

**Table-4:** Genotypic characterization of PVL-positive *S. aureus*.

Particulars	*spa*	*mecA*	*lukF-PV*
MSSA (n=191)	100	0	0
MRSA (n=57)	100	100	10.53

*S. aureus*=*Staphylococcus aureus*, MSSA=Methicillin-sensitive *Staphylococcus aureus*, MRSA=Methicillin-resistant *Staphylococcus aureus*, PVL=Panton-Valentine leukocidin

Similarly, wide variation is reported in the prevalence of PVL-positive *S. aureus* in humans. In India, in the study involving human isolates, the PVL gene was detected in 20 % of *S. aureus* isolates, predominantly in CA-*S. aureus* [16]. The prevalence of PVL-positive *S. aureus* varies widely between the countries with as low as 0.9% in Korea to a striking 97% in the United States [17].

The *spa* typing is considered as a frontline tool for the study of the short-term epidemiology of *S. aureus* [7]. The *spa* typing of the 6 PVL-positive isolates revealed five *spa* types in the local population (Table-5). The *spa* types t657, t1839, and t2526 are associated with emerging human MRSA epidemic clones of India [16]. It is plausible that these isolates could be of human origin. Recently, an outbreak and its successful management of bovine mastitis caused by New York/Japan (NJC) clone of Community Associated-MRSA were reported from Japan [18]. This study highlights the risk of emergence of new MRSA strains in dairy herd and an effective procedure against the spread of MRSA need to be based on molecular epidemiology studies.

**Table-5:** Summary of *spa* types found in PVL-positive isolates.

*spa* type	Number of repeats	Repeat succession	Number of isolates	Species	

				Cow	Buffalo
t657	8	26-23-13-21-17-34-33-34	1	0	1
t1839	10	26-23-13-21-17-34-34-34-33-34	2	2	0
t2526	10	07-12-21-17-13-13-13-34-33-13	1	1	0
t7286	7	07-16-12-23-02-34-34	1	0	1
t7684	7	07-23-21-34-34-33-34	1	1	0
Total isolates			6	4	2

PVL=Panton-Valentine leukocidin

The *spa* types t7286 and t7684 found in this study have been reported from the bovine milk in a study from Southern India [14]. This emphasizes that there is a need to understand the pathogenic role and significance of these strains in bovine mastitis.

The distribution of resistance to antimicrobials of the PVL-positive isolates is presented in Table-3. All the isolates were multidrug resistant by virtue of being resistant to methicillin. A high proportion of PVL-positive isolates were also resistant to ciprofloxacin, gentamicin, co-trimoxazole, and tetracycline which are common with MRSA of human origin. These antimicrobial agents are classified as critically important antimicrobials by the World Health Organization [7,11]. All the isolates were sensitive to chloramphenicol, linezolid, and pristinamycin.

In this study, the PVL-positive *S. aureus* was detected in raw pooled fresh milk sold at the different dairy outlets. The peri-urban area of Jabalpur is dotted with a large number of dairy farms with milking done exclusively by hand method. It is very likely that the contamination of milk through milkers’ could be a potential source of PVL-carrying *S. aureus* isolates. Given the PVL is phage-encoded virulence factor associated with CA-MRSA, the presence of such strains close to the consumer is of great public health concern.

## Authors’ Contributions

NS and VS contributed in the conception/design of the work. NS and AS conducted the work. AN and AKR assisted during the work. NS and AS prepared and corrected the manuscript. All the authors have read and approved the final manuscript.
